# An Inducible System for Rapid Degradation of Specific Cellular Proteins Using Proteasome Adaptors

**DOI:** 10.1371/journal.pone.0152679

**Published:** 2016-04-04

**Authors:** Shameika R. Wilmington, Andreas Matouschek

**Affiliations:** 1 Department of Molecular Biosciences, Northwestern University, Evanston, IL, United States of America; 2 Department of Molecular Biosciences, The University of Texas at Austin, Austin, TX, United States of America; University of Pittsburgh, UNITED STATES

## Abstract

A common way to study protein function is to deplete the protein of interest from cells and observe the response. Traditional methods involve disrupting gene expression but these techniques are only effective against newly synthesized proteins and leave previously existing and stable proteins untouched. Here, we introduce a technique that induces the rapid degradation of specific proteins in mammalian cells by shuttling the proteins to the proteasome for degradation in a ubiquitin-independent manner. We present two implementations of the system in human culture cells that can be used individually to control protein concentration. Our study presents a simple, robust, and flexible technology platform for manipulating intracellular protein levels.

## Introduction

There are many reasons to control protein concentrations artificially, for example to study complex biological systems without genetic manipulation or to elucidate protein function. The most common way to adjust protein concentrations artificially is by regulating protein synthesis. However, long-lived proteins persist after their synthesis has stopped, decaying only by dilution as cells grow and divide, which makes it difficult to modulate their abundance. Protein concentrations in the cell are a function of their rates of synthesis and degradation, so another way to manipulate protein abundance is by altering protein degradation. Most eukaryotic intracellular protein degradation is controlled by the ubiquitin proteasome system (UPS), which tunes the concentrations of hundreds of regulatory proteins [1]. Proteins are targeted to the proteasome by a degradation signal, or degron, that has two components: a proteasome-binding tag in the form of polyubiquitin chains and a proteasomal initiation region [2]. Degradation is regulated mainly by the covalent attachment of polyubiquitin chains, which serves as the proteasome-binding tag. The polyubiquitin chains are recognized by proteasome receptors and degradation initiates at a disordered region in the substrate called an initiation site [2]. The protein is then threaded into the proteolytic chamber where it is hydrolyzed into short peptides [1, 3].

Recruitment of a target protein to a ubiquitin ligase is usually sufficient to mediate its ubiquitination and several methods have been developed to control ubiquitination in this manner [4–6]. For example, bifunctional proteolysis targeting chimeras (PROTACs) are small molecules that bind to both the target protein and a specific E3 (refs [7, 8]). The PROTAC recruits the target protein to the E3 where it is ubiquitinated and routed to the proteasome for degradation. Related strategies direct the E3 ligase to the target protein through fusion proteins in which a truncated ligase or a ligase subunit is fused to an affinity domain that recognizes the target protein [9–13]. The target is again ubiquitinated and degraded by the proteasome.

In another set of approaches, the stability of the target protein is modulated through a destabilizing domain (DD) that is fused to the protein. The DD interacts with the cellular protein quality control system, leading to degradation by the proteasome, most likely after ubiquitination. Mutated forms of FKBP [14, 15], FRB [16] and DHFR [17, 18] domains, or a bacterial dehalogenase domain (Halo-Tag protein) [19] have all been used as DDs. A small molecule ligand or temperature then either inhibit or activate the DD and tune the stability of the entire protein. In an elegant variation, a degron is fused to the C terminus of the target protein, together with a viral protease that cleaves the degron immediately from the target protein leaving it untagged and stable. Small molecule inhibitors of the viral protease stabilize the full-length fusion protein so that the C-terminal degron induces the degradation of the entire protein [20].

Ubiquitin plays a role in many cellular processes other than proteasomal degradation and its regulation is complex and poorly understood. Interfering with ubiquitination networks can affect the many cellular pathways it controls, thereby leading to unintended pleiotropic effects on cells [21–23]. Some proteins are ubiquitinated but not degraded, while others are degraded by the proteasome yet not ubiquitinated [21, 24]. Therefore, we have developed a method to control protein degradation independent of the ubiquitination process.

In yeast, localizing a protein directly to the proteasome can lead to its degradation [25]. *In vitro*, it is possible to target proteins indirectly to the proteasome through a binding partner that contains a ubiquitin tag [26]. The binding partner with the ubiquitin tag serves as an adaptor that shuttles the target protein to the proteasome by interacting with the target and proteasome simultaneously. The proteasome can then initiate degradation at a disordered region in the target protein and digest it. Such adaptor proteins exist physiologically in the form of UbL (ubiquitin-like)-UBA (ubiquitin-associated) proteins [27]. The UbL domain shares homology with ubiquitin and binds to receptors on the proteasome, whereas the UBA domain recognizes ubiquitin chains on the target protein [28–30]. It is possible to design artificial proteasome adaptors that function in mammalian cells similarly to the UbL-UBA proteins but recognize specific proteins in lieu of ubiquitin chains. Thus we can manipulate the concentrations of specific cellular proteins by targeting them to the proteasome indirectly.

Methods have been developed to regulate complex formation by using chemical inducers of dimerization (CID) that control the interaction of two proteins by serving as a bridging ligand. The best established of these systems is based on a FK506 binding protein (FKBP12) and FKBP12-rapamycin-binding protein (FRB), which interact only in the presence of the small molecule rapamycin [4, 31]. FKBP12 is a 12 kDa cytosolic protein and FRB is an 11 kDa domain derived from mammalian target of rapamycin (mTor). The FKBP-FRB complex forms quickly and tightly in the presence of rapamycin, which has a high (nanomolar) affinity for both proteins [32]. The FKBP-rapamycin-FRB system has been used previously to control protein activity by modulating subcellular protein localization or activity. In different implementations, rapamycin targets a protein directly to a modified proteasome particle for degradation in yeast [25], sequesters proteins to cellular compartments [33], and controls complex formation to activate signal transduction cascades [34].

Here we asked whether it is possible to use an inducible dimerization system to control the cellular abundance of individual proteins using proteasome adaptors that shuttle otherwise stable proteins directly to the proteasome. The interaction of the adaptor with the target protein is controlled by CIDs. The systems described here differ from current technologies in multiple ways. First, the adaptors bind to the proteasome through ubiquitin-like domains bypassing the cellular ubiquitination machinery. Avoiding the ubiquitination step also makes it possible to fine tune degradation so that the concentration of the target protein can be tightly controlled. Second, the adaptor system as implemented here explicitly incorporates a proteasome initiation region, which makes it possible to degrade small compact proteins such as Green Fluorescent Protein (GFP). Third, the approach is versatile and can be implemented with orthologous chemically inducible dimerization systems, in principle making it possible to control the concentrations of several proteins simultaneously.

## Results and Discussion

### Construction of a proteasome adaptor and target protein

To test whether artificial proteasome adaptors can be used to control the cellular concentrations of specific proteins, we chose GFP as the target protein. GFP has been widely used to follow cellular protein concentrations [35, 36]. It is a stable, long-lived protein with a half-life of more than one day in mammalian culture cells [37] and it has proven challenging to degrade [10, 38, 39].

**Fig 1:**
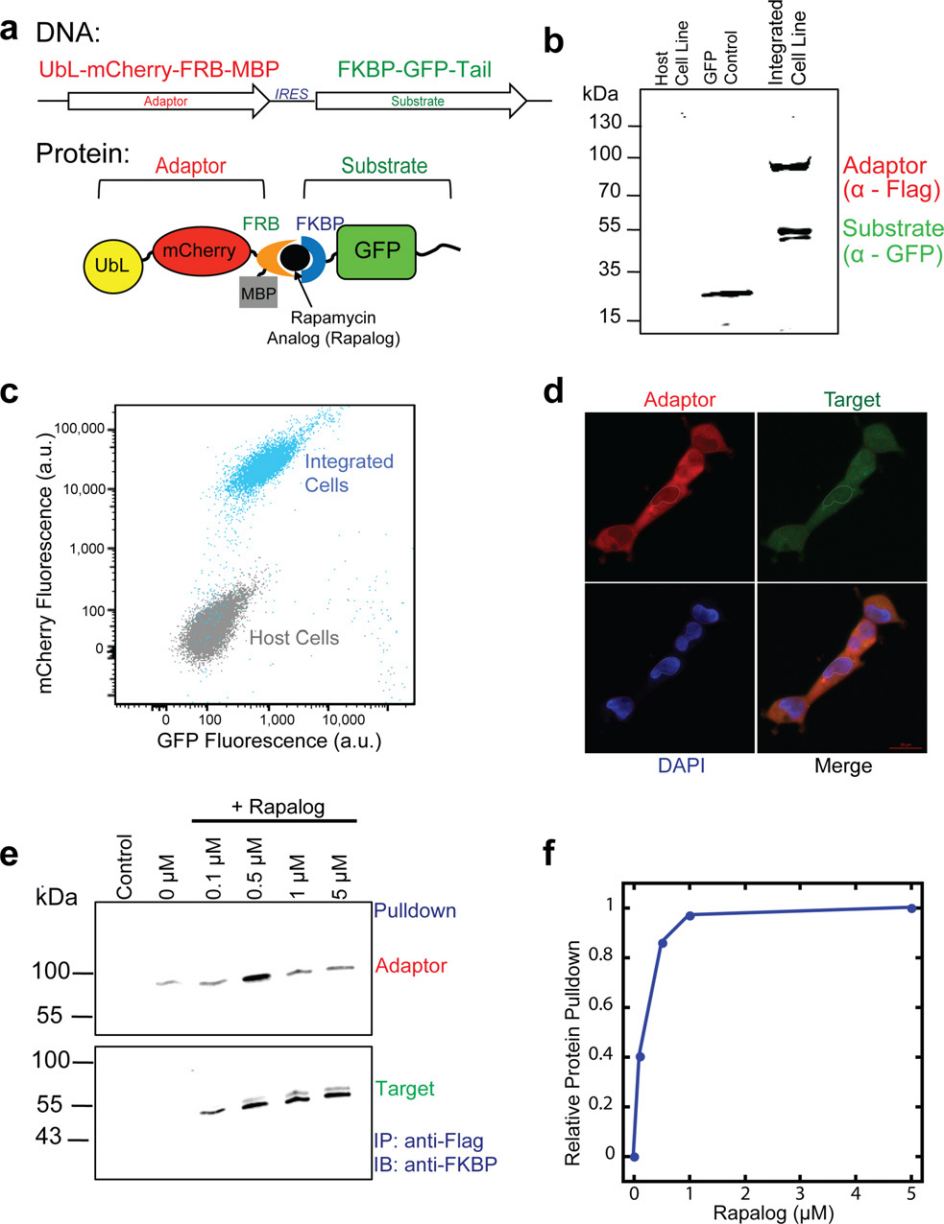
The inducible degradation cassette based on rapalog-inducible CID is stably integrated and expressed in mammalian HEK293 cells. (a) Schematic representation of the inducible degradation cassette at the DNA and protein level. Two proteins, the proteasome adaptor and the target protein, are expressed from a bicistronic plasmid with the coding regions separated by an internal ribosome entry site (IRES). The adaptor protein consists of a ubiquitin-like domain (UbL) followed by mCherry, a FRB domain, and MBP. The target protein consists of FKBP followed by the target protein GFP and a disordered region (tail). (b) Cell lysates were immunoblotted with antibodies against the indicated proteins; adaptor (α-Flag) and substrate (α-GFP). Lane 1 contains lysate from host cells; lane 2 contains lysate from cells expressing a GFP control gene inserted after the IRES; lane 3 contains lysate from cells with the inducible degradation cassette stably integrated into their genome and expressing both the adaptor (UbL-mCherry-FRB-MBP) and target protein (FKBP-GFP-tail). (c) Flow cytometry bivariate plot of host cells (gray) and cells with the integrated inducible degradation cassette (blue). X-axis shows GFP fluorescence and the Y-axis shows mCherry fluorescence for individual cells. (d) Fluorescence microscopy confirmed expression of the adaptor (mCherry) and target (GFP) expression in both the nucleus and cytoplasm. Scale bar, 20 μM. (e) Immunoprecipation of the Flag-tagged Adaptor protein (UbL-mCherry-FRB) with the GFP Substrate (FKBP-GFP-35) at different concentrations of rapalog. Lysates were immunoblotted with antibodies against the indicated proteins; adaptor (α-Flag) and substrate (α-FKBP). Substrate is only pulled down in the presence of rapalog. (f) Quantification of immunoprecipiation from (e) but with relative protein pull down normalized to the amount of precipitated protein at 5 μM rapalog.

We constructed an artificial proteasome adaptor by fusing the UbL domain of the human protein Rad23b [30] as the proteasome-binding tag to the N terminus of the human FRB domain. We added three more modifications to the adaptor: we inserted the red fluorescent protein mCherry [40] between the UbL and FRB domains to allow us to monitor abundance of the adaptor protein, we fused a maltose-binding domain to the C terminus of the FRB domain to stabilize the adaptor protein in cells [41], and we introduced a mutation into the FRB domain [16, 42] (equivalent to T2098L in full-length mTOR) to allow it to interact with a derivative of rapamycin (AP21967, MaRap or rapalog) [16, 42]. Rapalog no longer binds to endogenous mTOR and thus lacks rapamycin’s effect on cell proliferation through inhibition of the mTOR kinase. The final proteasome adaptor then consisted of an N-terminal UbL domain, followed by mCherry, the modified FRB domain, and a C-terminal MBP domain (UbL-mCherry-FRB-MBP; Fig 1A). We designed the adaptor to lack disordered regions such that itself escapes degradation and remains stable [26].

To allow the target protein, GFP, to interact with the proteasome adaptor, we fused it to the C terminus of human FKBP12. Effective degradation of a protein requires the presence of a disordered region that allows the proteasome to engage the protein and to initiate degradation [2, 26, 43, 44]. Therefore we fused a 35 amino acid disordered region or tail derived from yeast cytochrome *b*_2_ that has previously been shown to support proteasome initiation [43, 44] to the C terminus of GFP to yield FKBP-GFP-tail (Fig 1A).

We expressed both adaptor and target in a bicistronic expression vector from a CMV promoter in HEK293 cells. The coding region for the adaptor was separated from that of the target protein by an Internal Ribosome Entry Site (IRES) derived from the encephalomyocarditis virus (EMCV) [45] (Fig 1A). The vector was then integrated into the genome of HEK293 cells (Flp-In™ 293). Both the adaptor and substrate proteins were expressed and accumulated as verified by SDS-PAGE and western blotting (Fig 1B). The proteins were easily detected by flow cytometry (Fig 1C) and were colocalized throughout the cytoplasm and nucleus of all cells (Fig 1D). The abundance of the target was reduced relative to the adaptor in this expression system as is commonly observed for coding regions downstream of an IRES [46]. Rapalog induced the association of FRB and FKBP domains [31, 32] and led to the interaction of the proteasome adaptor and target protein. Immunoprecipitation of the adaptor protein yielded target protein, but only in the presence of rapalog (Fig 1E and 1F).

### Dose-dependent depletion of target protein but not adaptor

**Fig 2:**
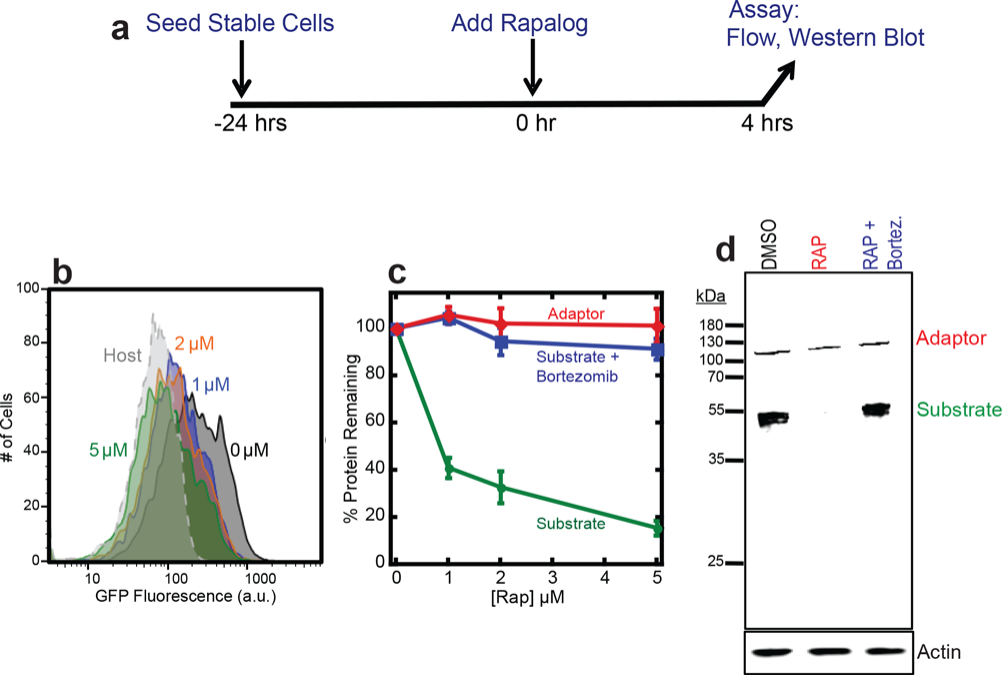
Dose-dependent degradation of the target protein. (**a**) Experimental design. HEK293 cells co-expressing the adaptor (UbL-mCherry-FRB-MBP) and target (FKBP-GFP-tail) were seeded 24 hours in advance. Degradation was initiated by the addition of rapalog and cells were incubated for four hours. The amount of target protein remaining in cells was assayed by flow cytometry of intact cells, or by western blotting of cell extracts. (**b**) Measurement of target protein abundance in cells by flow cytometry. The graph shows a histogram of GFP fluorescence of cells incubated with 0 μM (Black), 1 μM (Blue), 2 μM (Orange), 5 μM (Green) rapalog for four hours, as well as host control cells (Gray). (**c**) Quantification of adaptor and target protein abundance after incubation with CID or CID and proteasome inhibitor. The graph plots the average median cellular fluorescence as measured in (b) relative to the median GFP cellular fluorescence immediately after the addition of CID. Substrate: GFP fluorescence, adaptor: mCherry fluorescence. Data are presented as the average from experiments performed in triplicate. (**d**) Measurement of target protein abundance at 5 μM rapalog as in (c) of cell extracts by western blotting with antibodies against anti-Flag to detect the adaptor protein and anti-GFP to detect the target protein. As a loading control, actin levels were detected with antibodies against actin. DMSO: cells incubated with DSMO only; RAP: cells incubated with 5 μM rapalog; RAP+Bortez.: cells incubated with 5 μM rapalog and 1 μM bortezomib.

Next we tested whether the association of the target protein with the UbL-FRB adaptor led to the target protein’s degradation. We incubated HEK293 cells expressing adaptor and target with 1, 2, or 5 μM rapalog or mock-treated with DMSO for four hours and measured cell fluorescence by flow cytometry (Fig 2A). The green fluorescence intensity reporting the presence of the GFP target protein decreased almost completely to background level after incubation with 5 μM rapalog (Fig 2B), whereas the red fluorescence representing the mCherry-labeled adaptor remained constant (Fig 2C). The loss of green fluorescence was due to the complete degradation of the GFP target as SDS PAGE analysis of cell extracts followed by western blotting did not reveal any remaining GFP protein fragments (Fig 2D). Degradation was by the proteasome because adding the proteasome inhibitor bortezomib restored target protein abundance to the level seen in the absence of rapalog (Fig 2D).

The extent of depletion of the target protein depended on the concentration of rapalog added to the cells and increasing amounts of rapalog led to lower amounts of target protein accumulating (Fig 2B and 2C). Approximately 60% of the GFP target was depleted by incubation with 1 μM rapalog, whereas the majority (~90%) of the GFP target protein was depleted by incubation with 5 μM rapalog (Fig 2B and 2C). Thus, inducing dimerization of a proteasome adaptor and target made it possible to tune the accumulation of an otherwise stable protein in HEK293 cells.

### Degradation of the target protein is rapid

**Fig 3:**
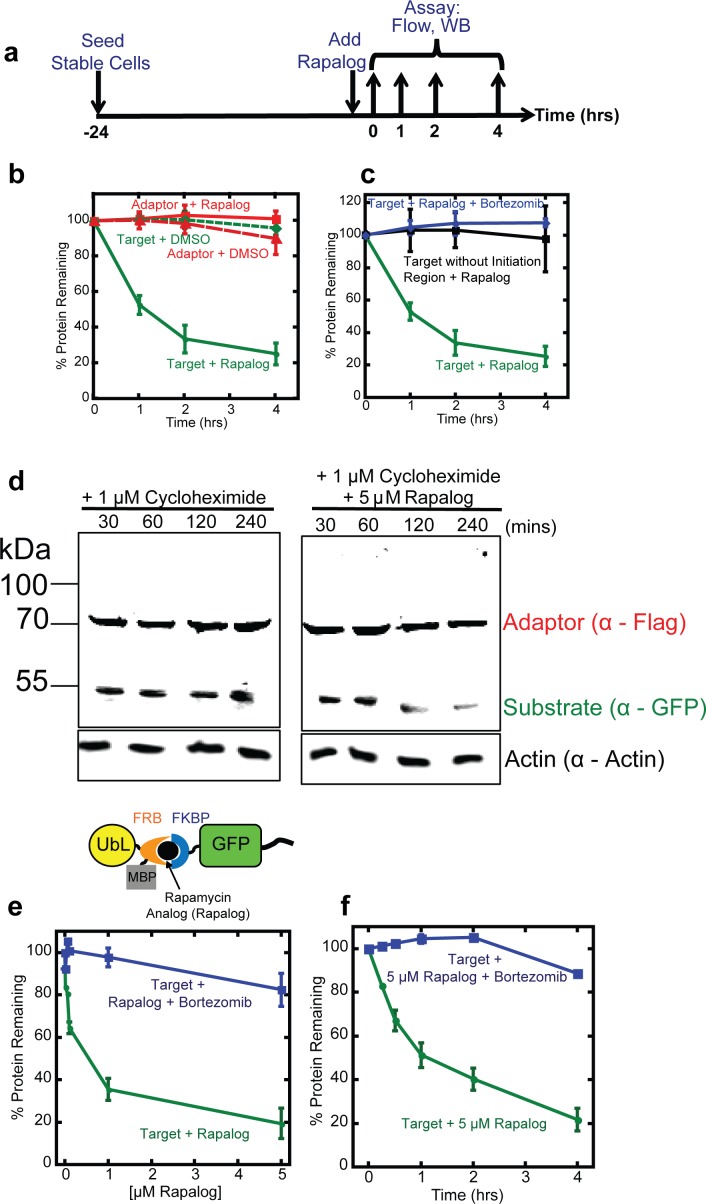
Rapalog-induced target degradation. (**a**) Experimental design. HEK293 cells co-expressing the adaptor (UbL-mCherry-FRB-MBP) and substrate (FKBP-GFP-tail) were seeded 24 hours prior to drug treatment. Degradation was initiated by the addition of 5 μM rapalog and samples were collected at the indicated timepoints. The amount of target protein remaining in cells was assayed by flow cytometry of intact cells or by western blotting of cell extracts. (**b**) Quantification of adaptor and target protein abundance after incubation with CID or DMSO. The graph plots the average median cellular fluorescence relative to the median GFP or mCherry cellular fluorescence immediately after the addition of CID. Data are presented as the average median fluorescence intensity ± SEM calculated from experiments performed in triplicate. (**c**) Quantification of adaptor, target, and target without disordered region protein abundance after incubation with CID or CID and proteasome inhibitor. The graph plots the average median cellular fluorescence relative to the median GFP or mCherry cellular fluorescence immediately after the addition of CID, which was set to 100%. Data are presented as the average median fluorescence intensity ± SEM calculated from experiments performed in triplicate. (**d**) Measurement of target protein abundance at indicated time points after treatment with 1μM cycloheximide (left panel) or 1μM cycloheximide and 5μM rapalog (right panel). Proteins were detected by western blotting with antibodies against anti-Flag to detect the adaptor protein and anti-GFP to detect the target protein. Actin, detected by anti-actin antibodies, was used as a loading control. (**e**) Quantification of the compact adaptor (UbL-FRB-MBP) and substrate (FKBP-GFP-tail) in HEK293 cells after incubation with different amounts of rapalog for 4 hours. The data are plotted as in (**b**) and (**c**). (**f**) Quantification of the compact adaptor (UbL-FRB-MBP) and substrate (FKBP-GFP-tail) in HEK293 cells over time after addition of 5 μM rapalog. The data are plotted as in (**b**) and (**c**).

We then determined how rapidly the target protein became depleted after the addition of rapalog. We incubated HEK293 cells expressing the adaptor and substrate with 5 μM rapalog and measured their fluorescence over time by flow cytometry (Fig 3A). The green fluorescence intensity, which reports the abundance of target protein, decreased with a half-life of about 1 hour (Fig 3B and 3C), which is significantly shorter than the reported half-life for GFP in mammalian cells (t_1/2_ = 26 hours) [37]. The red fluorescence intensity of the adaptor protein remained constant. Both green (target) and red (adaptor) fluorescence remained constant during mock treatment with DMSO (Fig 3B) and the proteasome inhibitor bortezomib inhibited target degradation in the presence of rapalog (Fig 3C).

Target protein abundance decreased upon rapalog addition because its half-life decreased. We estimated target protein half-life in cells by inhibiting protein synthesis with cycloheximide and following protein abundance in the presence or absence of rapalog. To this end, we treated cells expressing adaptor and target protein with 1μM cycloheximide and either 5 μM rapalog or DMSO. At different time points, we took samples and estimated protein levels by SDS PAGE and western blotting. The GFP target protein in cells treated with rapalog disappeared over time, whereas the adaptor protein remained largely stable (Fig 3D). Both target protein and adaptor remained stable in cells treated with vehicle only (Fig 3D).

Protein degradation in mammalian cells can be even faster [47–49] than the adaptor-induced degradation of GFP observed here. Indeed, GFP with a UbL domain directly fused to its N terminus and a tail at its C terminus (UbL-GFP-tail) is degraded with a half-life of less than 15 minutes in HEK293 cells (not shown). Adaptor mediated degradation may be slower because the CID has to diffuse into the cell and degradation requires three components present at relatively low concentrations to encounter each other. It is also possible that degradation of a protein presented to the proteasome by an adaptor molecule is intrinsically slower than degradation of a ubiquitinated protein.

Efficient proteasomal degradation *in vitro* and *in vivo* (yeast) requires the presence of a disordered sequence (or initiation region) in the substrate protein to allow the proteasome to engage the substrate [2, 43, 48]. The presence of disordered regions correlates with shorter half-lives of natural proteins [48, 49]. Thus including an effective proteasome initiation region in the target construct should make the inducible degradation system more effective. Hence, we explicitly included a proteasome initiation region in the target construct by including a disordered sequence (tail) at its C terminus. Removing the tail prevented degradation of the target protein even in the presence of rapalog (Fig 3C). The initiation region by itself did not lead to degradation (Fig 3B); the target protein is only degraded in the presence of proteasome adaptor, initiation region and rapalog (Fig 3B–3D). Thus providing the two necessary components of a degron, the proteasome-binding tag on the adaptor and the initiation region on the target results in rapid degradation of the substrate in the presence of the CID. The fact that the inducible degradation system described here is able to deplete GFP, which is notoriously hard to unfold and degrade [10, 38, 39], suggests that it should be effective for a wide range of target proteins.

### Compact proteasome adaptors

Fluorescent proteins, even optimized proteins such as mCherry, can be toxic to cells [40, 50]. Therefore, we tested whether the rapalog-dependent proteasome adaptor remained effective after removing the mCherry domain. We fused the Rad23Bb UbL domain directly to FRB-MBP (creating UbL-FRB-MBP; Fig 3E) and integrated the new adaptor together with the target protein into host cells as described above. Rapalog again induced degradation of the target proteins with roughly the same dose response as observed with the adaptor containing the mCherry domain. Incubation of the cells with 1 μM rapalog over four hours reduced target protein levels more than 60% and effectively all the protein was degraded during incubation with 5 μM rapalog (Fig 3E). Degradation was proteasome-dependent and abolished by the proteasome inhibitor bortezomib (Fig 3E and 3F). The compact adaptor depleted the target protein with a similar rate as the adaptor containing mCherry (Fig 3F). Thus, a compact proteasome adaptor could induce the degradation of a target protein when their dimerization was induced with rapalog.

### Plant hormone gibberellin can be used as a CID for target protein depletion

To test whether the efficacy of the rapalog-induced degradation system reflects its design principle and not some idiosyncratic property of one of its components, we developed a second inducible degradation system based on a different CID, the plant hormone gibberellin (GA_3_) (refs [34, 51]). Gibberellins regulate plant growth by inducing binding of the nuclear receptor Gibberellin Insensitive Dwarf 1 (GID1) to the DELLA domain of transcriptional regulators such as gibberellin insensitive (GAI) [34, 51]. The GID1 and GAI1 proteins only associate in the presence of the appropriate CID, here the gibberellin hormone GA_3_. GA_3_ diffuses through cell membranes poorly because it is negatively charged at neutral pH. However, esterification of its carboxylic acid group with an acetoxymethyl group (to generate GA_3_-AM) improves its membrane permeability significantly and intracellular esterases regenerate active GA_3_ after uptake [34].

**Fig 4:**
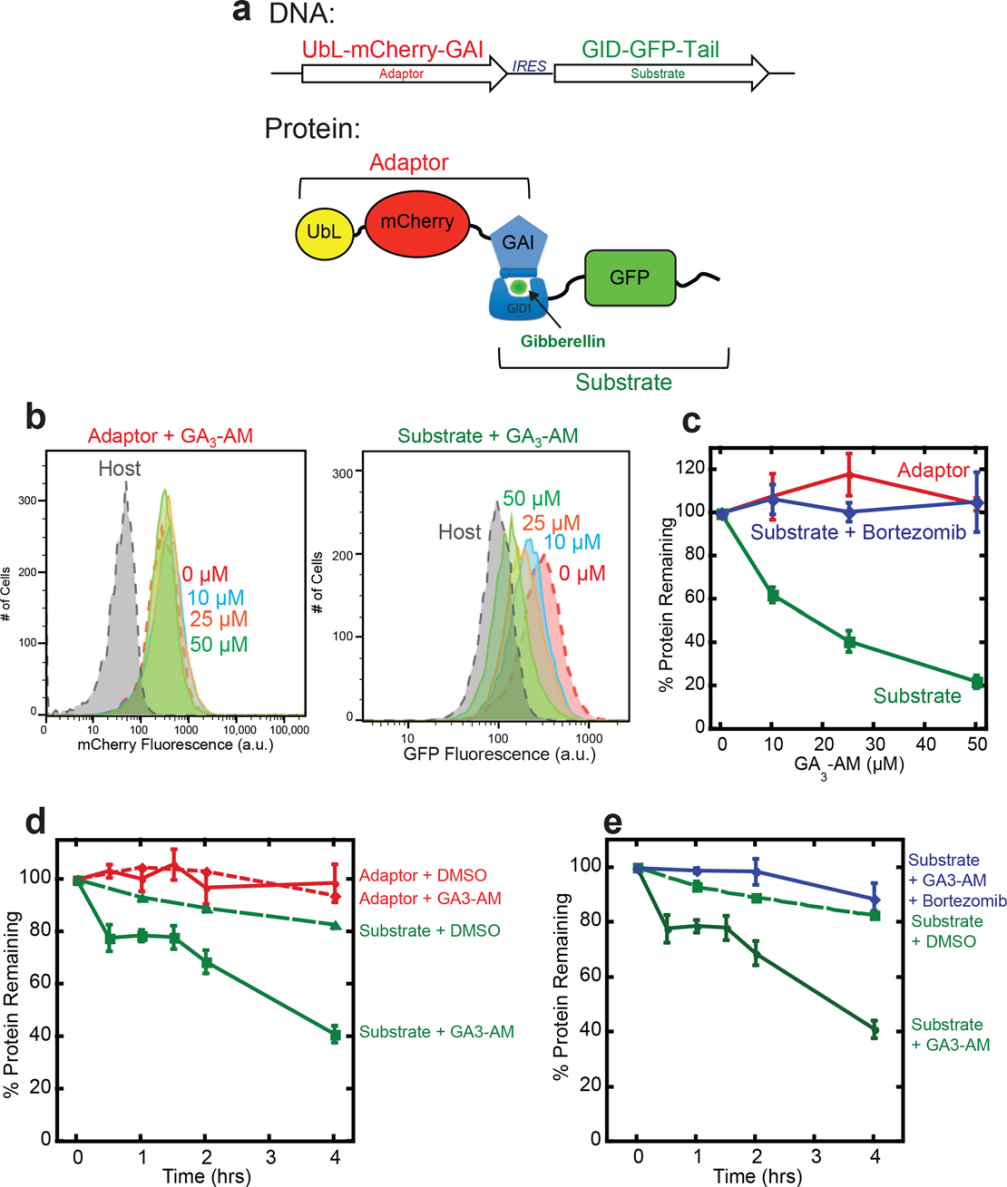
An inducible degradation system based on a gibberellin CID. (**a**) Schematic representation of the inducible degradation cassette at the DNA and protein level. Two proteins, the proteasome adaptor and the target protein, are expressed from a bicistronic plasmid with the coding regions separated by an internal ribosome entry site (IRES). The adaptor protein consists of a ubiquitin-like domain (UbL) followed by mCherry and a GAI domain. The target protein consists of GID followed GFP and a disordered region (tail). Gibberellin was esterified to produce a modified cell-permeable hormone, GA_3_-AM. (**b**) Flow cytometry histograms or (**c**) quantification of adaptor and target protein abundance after incubation with different concentrations of the CID, GA_3_-AM or GA_3_-AM and bortezomib for 24 hours. The graph plots the average median cellular fluorescence as relative to the median mCherry and GFP cellular fluorescence immediately after the addition of CID. Substrate: GFP fluorescence, adaptor: mCherry fluorescence. Data are presented as the average median fluorescence intensity ± SEM from an experiment performed in triplicate. (**d**) Quantification of adaptor and target protein abundance over time after incubation with the CID GA_3_-AM. The graph plots the average median cellular fluorescence relative to the median GFP or mCherry cellular fluorescence immediately after the addition of CID. Substrate: Target GFP fluorescence, adaptor: mCherry fluorescence. Data are presented as the average median fluorescence intensity ± SEM from an experiment performed in triplicate.

To develop an inducible degradation method based on gibberellin-mediated dimerization, we fused the GID1 domain to the N terminus of GFP and the same 35 amino acid disordered region used above to its C terminus (yielding GID1-GFP-tail). We created a proteasome adaptor by fusing the human Rad23b UbL domain to the N terminus of a GAI domain (amino acids 1–92 of GAI) through a mCherry domain (producing UbL-mCherry-GAI_1-92_). We again expressed target and adaptor using an IRES-containing bicistronic vector that was stably integrated into HEK293 Flp-In™ cells (Fig 4A). Both adaptor and substrate accumulated in cells and were readily detected by flow cytometry (Fig 4B) or fluorescence imaging (not shown). Incubation of the cells with 10 μM GA_3_-AM for 24 hours reduced cellular GFP fluorescence as measured by flow cytometry by 40%, suggesting that nearly half of the target protein was depleted (Fig 4B and 4C). Addition of 50 μM GA_3_-AM led to the depletion of nearly 80% of the target protein and higher GA_3_-AM concentrations did not lead to further depletion (Fig 4C). Incubation with DMSO vehicle alone did not affect target protein abundance and degradation was by the proteasome because the proteasome inhibitor bortezomib restored target protein levels (Fig 4C). Adaptor abundance remained unaffected over the entire GA_3_-AM concentration range as assessed by flow cytometry detecting mCherry fluorescence (Fig 4B and 4C). To determine how quickly GA_3_-AM induced GAI-GID1 degradation, we treated stable cells expressing adaptor and substrate with 50μM GA_3_ and monitored GFP fluorescence over time (Fig 4D). The GFP target protein depleted with a half-life of four hours, while the adaptor remained stable (Fig 4D). Neither GFP nor mCherry fluorescence changed over time in cells mock-treated with DMSO or with both GA_3_-AM and bortezomib (Fig 4D and 4E).

The gibberellin-induced dimerization system required a higher concentration of CID to induce degradation and the target protein depleted more slowly than observed for the rapalog-induced degradation system, showing a lag in the degradation kinetics. Gibberellin may be less available in cells, either because its alkylated derivative is slower to diffuse across the membrane or because the alkylated form is inefficiently converted back to GA_3_ inside cells. In addition, GID1 must undergo a conformational change after GA_3_ biding to interact with its dimerization partner GAI, and this process may proceed more slowly than the direct interaction of rapamycin with its binding partners. Nevertheless, proteasome adaptors that interacted with their target through gibberellin-inducible dimerization were able to control the degradation of our target protein effectively.

The proteins and CIDs of the gibberellin and rapamycin inducible dimerization systems are unrelated [34] yet both systems allowed proteasome adaptors to induce degradation of the target protein effectively. Thus, the inducible protein degradation by proteasome adaptors does not depend on the details of construction of the adaptor itself. This versatility should make it possible to control the abundance of multiple proteins, for example, by combining compatible inducible dimerization systems, such as the rapalog and gibberellin systems used here. The proteasome can respond robustly to increased load [52–55] so that it may be possible to expand the proteasome adaptor approach by multiplexing additional CID systems to allow the analysis of several proteins within a given process or in multiple pathways simultaneously.

## Conclusions

**Fig 5:**
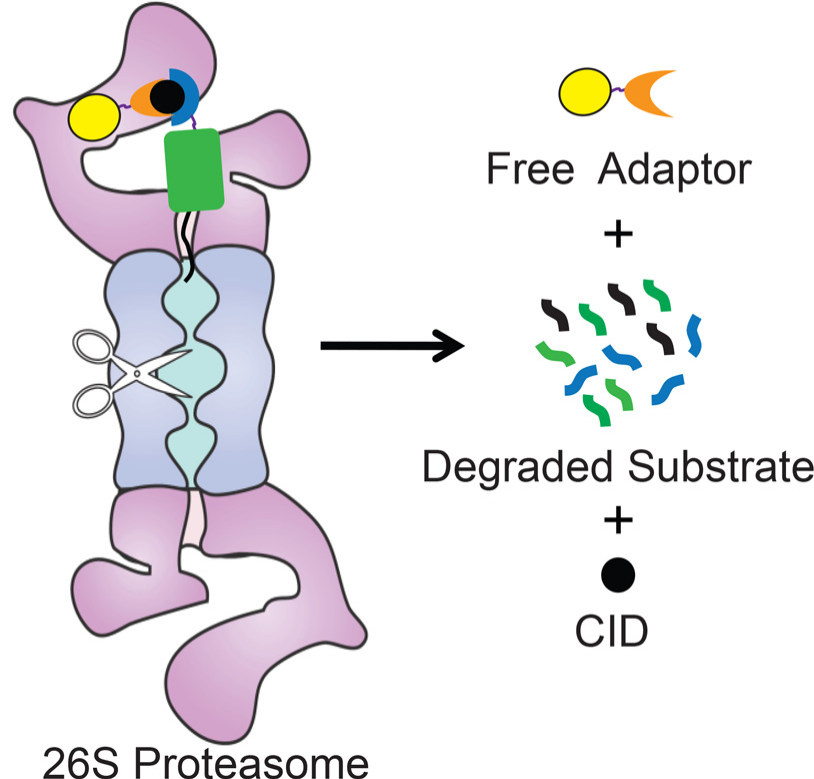
Model for Adaptor-CID-mediated degradation of target proteins. Schematic representation of adaptor-mediated degradation of a target protein by the proteasome. The proteasome is represented by the purple and blue shape, the approximate location of its proteolytic sites are indicated by scissors. A substrate, consisting of the target protein (green rectangle) fused to an interaction domain (blue crescent) and a proteasome initiation region (black line) is brought to the proteasome by a proteasome adaptor consisting of a UbL (yellow sphere) and a second interaction domain (orange crescent). Adaptor and substrate interact only in the presence of a CID (black sphere).

We describe a method to control the abundance of specific proteins by directly targeting them to the proteasome for degradation. This system is based on proteasome adaptors that bind to the proteasome and target protein simultaneously and feed the target to the proteasome for degradation (Fig 5). The adaptor itself escapes degradation and is able to recycle. The target protein is fused to an interaction domain that binds to the proteasome adaptor only in the presence of a chemical inducer of dimerization, and a disordered region that allows the proteasome to initiate degradation efficiently. This novel system provides a direct way to tune the abundance of a target protein concentration by rapidly depleting proteins from cells without the need for ubiquitination.

## Materials and Methods

### Plasmid construction

Constructs were generated by a combination of InFusion (Clontech), PCR, site-directed mutagenesis (Invitrogen), PCR-assembly and restriction-mediated cloning (NEB and Fermentas). All PCR-generated constructs were verified by sequencing analysis. Final constructs were expressed in the pcDNA5/FRT/TO vector (modified from Life Technologies) under the control of the human cytomegalovirus (CMV) enhancer-promoter. GAI and GID coding sequences were obtained from Addgene (Plasmid #37309 and #37306, respectively).

### Cell culture

Cell lines were cultured in DMEM (Gibco) supplemented with 10% FBS (Gibco) and 1% PenStrep (Gibco) at 37°C and 5% CO_2_ according to standard procedures. For stable expression of proteins, stable cell lines were generated using the HEK293 Flp-In^TM^ System (Life Technologies). Cells were transiently transfected with 1 μg of pcDNA5/FRT/TO vector containing cloned DNA + 9 μg of poG44 DNA using Lipofectamine 2000 (Invitrogen) followed by selection of stable clones with hygromycin B (200 μg/mL). Stable cell lines were confirmed by flow cytometry and Western blotting analysis.

### Flow cytometry and fluorescence microscopy

For dose-dependence and kinetic assays HEK293 Flp^TM^-In stable cells expressing Rapalog and Gibberellin-specific adaptor and target proteins were plated at 5 X 10^5^ cells per well of a 6-well plate and treated with Rapalog or Gibberellin (GA_3_) and (1 μM) bortezomib (LC Laboratories) at indicated concentrations or time points prior to analysis. Cells were washed with 1 mL of PBS (Gibco) and detached from wells using 500 μL of TrypLE-Express (Invitrogen) and quenched with 1 mL DMEM without phenol red containing 10% FBS and 1% Penstrep. Samples were analyzed using the BD Fortessa with 10,000 events typically represented. The resulting data was quantified using FlowJo. For microscopy cells were plated on a microscope dish and imaged using a Zeiss LSM 710 microscope 63X N.A. 1.4.

### Western blotting

HEK293 Flp^TM^ In stable cells were washed in PBS and lysed on ice in whole cell extract buffer (50 mM Tris, pH 8.0, 280 mM NaCL, 0.5% Nonidet P-40, 0.2mM EDTA, 2mM EGTA, 10% glycerol, 1 mM DTT, 2 mM PMSF, and protease inhibitor mixture set III, EDTA-free (Calbiochem) and 0.2 mM Sodium Orthovanadate) for 10 minutes. Cells were then centrifuged at 13,000 RPM 4°C for 10 minutes and the lysate was collected. Protein sample buffer was added to the lysate and heated for 5 min at 95°C. Cell lysates were separated by SDS-PAGE on a 10% Tris-Tricine gel. The blots were processed by standard procedures and probed with Flag (mouse, Sigma Cat# F9291), Living Colors eGFP (mouse, Clontech Cat# 632569), FKBP12 (rabbit, Abcam Cat# 2918) and actin (rabbit, Sigma Cat # A2066) antibodies. Following incubation with an IRDye 680 (Invitrogen Cat#1027681) or 800-conjugated secondary antibody (Rockland Immunochemicials Cat #610-132-121), the membranes were imaged using the Odyssey Infrared Imaging System (LI-COR). Results shown are representative of at least three independent experiments.

### Co-Immunoprecipitation

HEK293 Flp-In^TM^ stable cells were seeded at 3,000,000 cells per 10-cm2 dish. After 24 hours the media was replaced with DMEM/10% FBS/1% PenStrep containing rapalog (A/C Heterodimerizer from Clontech Catalog# 635056) at various concentrations, and incubated at 37°C with 5% CO_2_ for four hours. Media was aspirated and cells were washed and scraped from a 10-cm^2^ dish with 1 mL of cold phosphate-buffered saline (PBS). Lysates were prepared in whole cell extract (WCE) buffer following standard lysis procedures. Protein complexes were purified by overnight incubation with FLAG M2 affinity magnetic resin (Sigma Catalog# M8823) and washed with WCE buffer. Complexes were eluted from magnetic beads with 5-packaged gel volumes of 3X FLAG peptide (5 μg/μl) for 30 minutes while shaking at 4°C. Samples were placed in a magnetic separator to collect the beads. Supernatants were transferred to fresh tubes containing 5X sample buffer including 2-mercaptoethanol (BME). Proteins were then boiled at 95°C for 5 minutes. Samples were separated by sodium dodecyl sulfate-polyacrylamide gel electrophoresis (SDS-PAGE) and processed for immunoblotting. For immunoblotting, proteins were separated, transferred to nitrocellulose, probed with antibodies recognizing FLAG (Sigma) or FKBP (Abcam). Protein amounts were estimated by direct infrared fluorescence imaging (Odyssey LICOR Biosciences).

### Rapamycin derivatives

Rapalog AP21967 was supplied from Ariad Pharmaceuticals (MTA) as 250 μg of lyophilized powder. AP21967 was resuspended in 246 μL of 100% pure ethanol yielding a 1 mM stock. Rapalog A/C Heterodimerizer was (5 X 500μL Cat # 635056 and 5mgs Cat #635055) purchased from Clontech. 5mgs was dissolved in 747 μL of DMSO to produce a 192 mM stock.

### Chemical synthesis of gibberellin analog GA_3_-AM

Gibberellin acetoxymethyl (AM) was synthesized as previously described [34]. All reagents and solvents were supplied by commercial sources without further purification.

## References

[1] Finley D. Recognition and Processing of Ubiquitin-Protein Conjugates by the Proteasome. Annu Rev Biochem. 2009;78:477–513. 10.1146/annurev.biochem.78.081507.101607 19489727 PMC3431160

[2] Prakash S, Tian L, Ratliff KS, Lehotzky RE, Matouschek A. An unstructured initiation site is required for efficient proteasome-mediated degradation. Nat Struct Mol Biol 2004;11(9):830–7. 15311270 10.1038/nsmb814

[3] Tomko RJ, Hochstrasser M. Molecular architecture and assembly of the eukaryotic proteasome. Annu Rev Biochem. 2013;82(1):415–45. 23495936 10.1146/annurev-biochem-060410-150257 PMC3827779

[4] Rakhit R, Navarro R, Wandless TJ. Chemical biology strategies for posttranslational control of protein function. Chem Biol. 2014;21(9):1238–52. 10.1016/j.chembiol.2014.08.011 25237866 PMC4174368

[5] Schrader EK, Wilmington SR, Matouschek A. Making It Easier to Regulate Protein Stability. Chem Biol 2010;17(9):917–8. 10.1016/j.chembiol.2010.09.004 20851337 PMC2990474

[6] Buckley DL, Crews CM. Small-Molecule Control of Intracellular Protein Levels through Modulation of the Ubiquitin Proteasome System. Angew Chem Int Ed Engl. 2014;53(9):2312–30. 10.1002/anie.201307761 24459094 PMC4348030

[7] Sakamoto KM, Kim KB, Kumagai A, Mercurio F, Crews CM, Deshaies RJ. Protacs: chimeric molecules that target proteins to the Skp1-Cullin-F box complex for ubiquitination and degradation. Proc Natl Acad Sci USA. 2001;98(15):8554–9. 11438690 10.1073/pnas.141230798 PMC37474

[8] Bondeson DP, Mares A, Smith IED, Ko E, Campos S, Miah AH, et al Catalytic in vivo protein knockdown by small-molecule PROTACs. Nat Chem Biol. 2015. 10.1038/nchembio.1858 PMC4629852 26075522

[9] Portnoff AD, Stephens EA, Varner JD, Delisa MP. Ubiquibodies: Synthetic E3 Ubiquitin Ligases Endowed with Unnatural Substrate Specificity for Targeted Protein Silencing. J Biol Chem. 2014. 10.1074/jbc.M113.544825 PMC3953296 24474696

[10] Caussinus E, Kanca O, Affolter M. Fluorescent fusion protein knockout mediated by anti-GFP nanobody. Nat Struct Mol Biol. 2012;19(1):117–21. 10.1038/nsmb.2180 22157958

[11] Hatakeyama S, Watanabe M, Fujii Y, Nakayama KI. Targeted destruction of c-Myc by an engineered ubiquitin ligase suppresses cell transformation and tumor formation. Cancer Res. 2005;65(17):7874–9. 16140957 10.1158/0008-5472.CAN-05-1581

[12] Ma Y, Gu Y, Zhang Q, Han Y, Yu S, Lu Z, et al Targeted degradation of KRAS by an engineered ubiquitin ligase suppresses pancreatic cancer cell growth in vitro and in vivo. Mol Cancer Ther. 2013;12(3):286–94. 10.1158/1535-7163.MCT-12-0650 23288781

[13] Nishimura K, Fukagawa T, Takisawa H, Kakimoto T, Kanemaki M. An Auxin-Based Degron System for the Rapid Depletion of Proteins in Nonplant Cells. Nat Meth. 2009;6(12):917–22. 10.1038/nmeth.1401 19915560

[14] Chu BW, Banaszynski LA, Chen L-c, Wandless TJ. Recent progress with FKBP-derived destabilizing domains. Bioorg Med Chem Lett. 2008;18(22):5941–4. 10.1016/j.bmcl.2008.09.043 18815033 PMC2593907

[15] Maynard-Smith LA, Chen L-c, Banaszynski LA, Ooi AGL, Wandless TJ. A directed approach for engineering conditional protein stability using biologically silent small molecules. J Biol Chem. 2007;282(34):24866–72. 17603093 10.1074/jbc.M703902200 PMC3290522

[16] Stankunas K, Bayle JH, Gestwicki JE, Lin Y-M, Wandless TJ, Crabtree GR. Conditional protein alleles using knockin mice and a chemical inducer of dimerization. Mol Cell. 2003;12(6):1615–24. 14690613 10.1016/s1097-2765(03)00491-x

[17] Iwamoto M, Björklund T, Lundberg C, Kirik D, Wandless TJ. A General Chemical Method to Regulate Protein Stability in the Mammalian Central Nervous System. Chem Biol 2010;17(9):981–8. 10.1016/j.chembiol.2010.07.009 20851347 PMC2943492

[18] Dohmen RJ, Wu P, Varshavsky A. Heat-inducible degron: a method for constructing temperature-sensitive mutants. Science. 1994;263(5151):1273–6. 8122109 10.1126/science.8122109

[19] Neklesa TK, Tae HS, Schneekloth AR, Stulberg MJ, Corson TW, Sundberg TB, et al Small-Molecule Hydrophobic Tagging–Induced Degradation of HaloTag Fusion Proteins. Nat Chem Biol. 2011;7(8):538–43. 10.1038/nchembio.597 21725302 PMC3139752

[20] Chung HK, Jacobs CL, Huo Y, Yang J, Krumm SA, Plemper RK, et al Tunable and reversible drug control of protein production via a self-excising degron. Nature Chemical Biology. 2015;11(9):713–20. 10.1038/nchembio.1869 26214256 PMC4543534

[21] Komander D, Rape M. The ubiquitin code. Annu Rev Biochem. 2012;81:203–29. 10.1146/annurev-biochem-060310-170328 22524316

[22] Hanna J, Meides A, Zhang DP, Finley D. A ubiquitin stress response induces altered proteasome composition. Cell. 2007;129(4):747–59. 17512408 10.1016/j.cell.2007.03.042

[23] Marangos P, Carroll J. Securin regulates entry into M-phase by modulating the stability of cyclin B. Nat Cell Biol. 2008;10(4):445–51. 10.1038/ncb1707 18364698

[24] Erales J, Coffino P. Ubiquitin-independent proteasomal degradation. Biochim Biophys Acta. 2014;1843(1):216–21. 10.1016/j.bbamcr.2013.05.008 23684952 PMC3770795

[25] Janse DM. Localization to the Proteasome Is Sufficient for Degradation. J Biol Chem. 2004;279(20):21415–20. 15039430 10.1074/jbc.M402954200

[26] Prakash S, Inobe T, Hatch AJ, Matouschek A. Substrate selection by the proteasome during degradation of protein complexes. Nat Chem Biol 2009;5(1):29–36. 10.1038/nchembio.130 19029916 PMC2670781

[27] Schrader EK, Harstad KG, Matouschek A. Targeting proteins for degradation. Nat Chem Biol. 2009;5(11):815–22. 10.1038/nchembio.250 19841631 PMC4228941

[28] Wilkinson CR, Seeger M, Hartmann-Petersen R, Stone M, Wallace M, Semple C, et al Proteins Containing the UBA Domain are able to Bind to Multi-Ubiquitin Chains. Nat Cell Biol. 2001;3(10):939–43. 11584278 10.1038/ncb1001-939

[29] Elsasser S, Gali RR, Schwickart M, Larsen CN, Leggett DS, Müller B, et al Proteasome subunit Rpn1 binds ubiquitin-like protein domains. Nat Cell Biol. 2002;4(9):725–30. 12198498 10.1038/ncb845

[30] Rosenzweig R, Bronner V, Zhang D, Fushman D, Glickman MH. Rpn1 and Rpn2 coordinate ubiquitin processing factors at proteasome. J Biol Chem. 2012;287(18):14659–71. 10.1074/jbc.M111.316323 22318722 PMC3340268

[31] Choi J, Chen J, Schreiber SL, Clardy J. Structure of the FKBP12-rapamycin complex interacting with the binding domain of human FRAP. Science. 1996;273(5272):239–42. 8662507 10.1126/science.273.5272.239

[32] Banaszynski LA, Liu CW, Wandless TJ. Characterization of the FKBP.rapamycin.FRB ternary complex. J Am Chem Soc. 2005;127(13):4715–21. 15796538 10.1021/ja043277y

[33] Robinson MS, Sahlender DA, Foster SD. Rapid Inactivation of Proteins by Rapamycin-Induced Rerouting to Mitochondria. Dev Cell. 2010;18(2):324–31. 10.1016/j.devcel.2009.12.015 20159602 PMC2845799

[34] Miyamoto T, DeRose R, Suarez A, Ueno T, Chen M, Sun T-p, et al Rapid and orthogonal logic gating with a gibberellin-induced dimerization system. Nat Chem Biol. 2012;8(5):465–70. 10.1038/nchembio.922 22446836 PMC3368803

[35] Tsien RY. The green fluorescent protein. Annu Rev Biochem. 1998. 10.1146/annurev.biochem.67.1.509 9759496

[36] Remington SJ. Green fluorescent protein: a perspective. Protein Sci. 2011;20(9):1509–19. 10.1002/pro.684 21714025 PMC3190146

[37] Corish P, Tyler-Smith C. Attenuation of green fluorescent protein half-life in mammalian cells. Protein Eng. 1999;12(12):1035–40. 10611396 10.1093/protein/12.12.1035

[38] Nager AR, Baker TA, Sauer RT. Stepwise Unfolding of a β Barrel Protein by the AAA+ ClpXP Protease. J Mol Biol. 2011;413(1):4–16. 10.1016/j.jmb.2011.07.041 21821046 PMC3184388

[39] Martin A, Baker TA, Sauer RT. Protein unfolding by a AAA+ protease is dependent on ATP-hydrolysis rates and substrate energy landscapes. Nat Struct Mol Biol. 2008;15(2):139–45. 10.1038/nsmb.1380 18223658

[40] Shaner NC, Campbell RE, Steinbach PA, Giepmans BNG, Palmer AE, Tsien RY. Improved monomeric red, orange and yellow fluorescent proteins derived from Discosoma sp. red fluorescent protein. Nat Biotechnol. 2004;22(12):1567–72. 15558047 10.1038/nbt1037

[41] Scirè A, Marabotti A, Aurilia V, Staiano M, Ringhieri P, Iozzino L, et al Molecular strategies for protein stabilization: the case of a trehalose/maltose-binding protein from Thermus thermophilus. Proteins. 2008;73(4):839–50. 10.1002/prot.22114 18506781

[42] Liberles SD, Diver ST, Austin DJ, Schreiber SL. Inducible gene expression and protein translocation using nontoxic ligands identified by a mammalian three-hybrid screen. Proc Natl Acad Sci USA. 1997;94(15):7825–30. 9223271 10.1073/pnas.94.15.7825 PMC21513

[43] Fishbain S, Prakash S, Herrig A, Elsasser S, Matouschek A. Rad23 escapes degradation because it lacks a proteasome initiation region. Nat Comm. 2011;2:192–9. 10.1038/ncomms1194 PMC4069258 21304521

[44] Inobe T, Fishbain S, Prakash S, Matouschek A. Defining the geometry of the two-component proteasome degron. Nat Chem Biol. 2011;7(3):161–7. 10.1038/nchembio.521 21278740 PMC3129032

[45] Gurtu V, Yan G, Zhang G. IRES Bicistronic Expression Vectors for Efficient Creation of Stable Mammalian Cell Lines Biochem Biophys Res Commun. 1996:1–4. 10.1006/bbrc.1996.1795 8954121

[46] Mizuguchi H. IRES-Dependent Second Gene Expression Is Significantly Lower Than Cap-Dependent First Gene Expression in a Bicistronic Vector. Mol Ther. 2000;1(4):376–82. 10933956 10.1006/mthe.2000.0050

[47] Schwanhäusser B, Busse D, Li N, Dittmar G, Schuchhardt J, Wolf J, et al Global quantification of mammalian gene expression control. Nature. 2011;473(7347):337–42. 10.1038/nature10098 21593866

[48] Fishbain S, Inobe T, Israeli E, Chavali S, Yu H, Kago G, et al Sequence composition of disordered regions fine-tunes protein half-life. Nat Struct Mol Biol. 2015;22(3):214–21. 10.1038/nsmb.2958 25643324 PMC4351145

[49] van der Lee R, Lang B, Kruse K, Gsponer J, Sánchez de Groot N, Huynen MA, et al Intrinsically disordered segments affect protein half-life in the cell and during evolution. Cell Rep. 2014;8(6):1832–44. 10.1016/j.celrep.2014.07.055 25220455 PMC4358326

[50] Shemiakina II, Ermakova GV, Cranfill PJ, Baird MA, Evans RA, Souslova EA, et al A monomeric red fluorescent protein with low cytotoxicity. Nat Comm. 2012;3:1204. 10.1038/ncomms2208 23149748

[51] Murase K, Hirano Y, Sun T-p, Hakoshima T. Gibberellin-induced DELLA recognition by the gibberellin receptor GID1. Nature. 2008;456(7221):459–63. 10.1038/nature07519 19037309

[52] Wang X, Xu H, Ha S-W, Ju D, Xie Y. Proteasomal degradation of Rpn4 in Saccharomyces cerevisiae is critical for cell viability under stressed conditions. 2010;184(2):335–42. 10.1534/genetics.109.112227 19933873 PMC2828715

[53] Wang X, Xu H, Ju D, Xie Y. Disruption of Rpn4-induced proteasome expression in Saccharomyces cerevisiae reduces cell viability under stressed conditions. 2008;180(4):1945–53. 10.1534/genetics.108.094524 18832351 PMC2600933

[54] Radhakrishnan SK, Lee CS, Young P, Beskow A, Chan JY, Deshaies RJ. Transcription factor Nrf1 mediates the proteasome recovery pathway after proteasome inhibition in mammalian cells. 2010;38(1):17–28. 10.1016/j.molcel.2010.02.029 20385086 PMC2874685

[55] Xu H, Fu J, Ha S-W, Ju D, Zheng J, Li L, et al The CCAAT box-binding transcription factor NF-Y regulates basal expression of human proteasome genes. 2012;1823(4):818–25. 10.1016/j.bbamcr.2012.01.002 22285817

